# Embodied and embedded ecological rationality: A common vertebrate mechanism for action selection underlies cognition and heuristic decision-making in humans

**DOI:** 10.3389/fpsyg.2022.841972

**Published:** 2022-11-17

**Authors:** Samuel A. Nordli, Peter M. Todd

**Affiliations:** ^1^Cognitive Science Program, Indiana University, Bloomington, IN, United States; ^2^Department of Psychological and Brain Sciences, Indiana University, Bloomington, IN, United States

**Keywords:** ecological rationality, vertebrate motor control, cortico-basal ganglia-thalamo-cortical loop, habit formation, exaptation

## Abstract

The last common ancestor shared by humans and other vertebrates lived over half a billion years ago. In the time since that ancestral line diverged, evolution by natural selection has produced an impressive diversity—from fish to birds to elephants—of vertebrate morphology; yet despite the great species-level differences that otherwise exist across the brains of many animals, the neural circuitry that underlies motor control features a functional architecture that is virtually unchanged in every living species of vertebrate. In this article, we review how that circuitry facilitates motor control, trial-and-error-based procedural learning, and habit formation; we then develop a model that describes how this circuitry (embodied in an agent) works to build and refine sequences of goal-directed actions that are molded to fit the structure of the environment (in which the agent is embedded). We subsequently review evidence suggesting that this same functional circuitry became further adapted to regulate cognitive control in humans as well as motor control; then, using examples of heuristic decision-making from the ecological rationality tradition, we show how the model can be used to understand how that circuitry operates analogously in both cognitive and motor domains. We conclude with a discussion of how the model encourages a shift in perspective regarding ecological rationality’s “adaptive toolbox”—namely, to one that views heuristic processes and other forms of goal-directed cognition as likely being implemented by the same neural circuitry (and in the same fashion) as goal-directed action in the motor domain—and how this change of perspective can be useful.

## Introduction

The field of ecological rationality (e.g., Todd et al., 2012) is predicated on the assumption that any answer to questions regarding the “rationality” of a given animal’s behavior must necessarily include a proper accounting of (1) the evolved structure of the animal that exhibited the behavior, (2) the structure of the environment in which that behavior occurred, and (3) the structure of the environments in which the animal’s ancestral species evolved (if structural differences between present and past environments are plausible). Although researchers in ecological rationality have often restricted their analyses to the structure of a decision-maker’s mind (setting aside the mind’s implicit dependence on the structure of the brain/body), work in this tradition ideally seeks to understand behavior and cognition from the broadest relevant vantage point—which naturally includes the embodied perspectives and embedded contexts of thinking and acting agents. Ecology is the study of interactions between organisms and their environments; indeed, *ecological* rationality is so named to specifically call out those interactions, and hence already implies an embodied and embedded perspective. Moreover, von Uexküll’s (1957) inherently embodied and embedded concept of the Umwelt has been used explicitly within the ecological rationality community for years (e.g., Todd and Gigerenzer, 2012), and so the phrase “embodied and embedded ecological rationality” may admittedly seem redundant to some readers; however, we use it to draw attention to this connection, because others have criticized work in ecological rationality for overemphasizing environmental structure while underemphasizing the species-specific (and specificity-dependent) nature of decision-making environments (for further discussion/debate, see Felin et al., 2017; Chater et al., 2018; Felin and Koenderink, 2022).

This paper seeks to emphasize that the full extent and import of an agent’s embodiment and embeddedness may be obscured by the lenses through which that agent’s behavior and cognition are understood and described. For instance, although the heuristics and biases program (e.g., Tversky and Kahneman, 1974) takes inspiration from Simon’s (1955) concept of bounded rationality, one of the fundamental insufficiencies of that program (from an ecological rationality perspective) is the failure to fully account for natural selection, leading to an impoverished understanding and description of behavioral data. In the heuristics and biases view, the predictable use and failure of specific heuristics in certain contexts is seen as evidence of human irrationality and presented as the conclusion of a cautionary tale. From an ecological rationality perspective, the same findings are instead a starting point for further inquiry—indicative of how underlying cognitive and behavioral mechanisms typically function, as well as providing insight into why those mechanisms evolved to operate that way.

The ecological rationality tradition has also not been immune to such perspectival limitations. Applying the same general criticism through the lens of a Marrian perspective (Marr, 1982), the ecological rationality literature has historically tended to restrict itself to computational and algorithmic levels of analysis (Gallese et al., 2020), focusing on the structure of environmental problems and the algorithmic tools used to solve them, while tending to eschew consideration of how those tools are constructed and implemented in terms of their underlying neuroanatomy and physiology. In fairness, many psychologists and cognitive scientists will openly admit that they ignore the brain in their thinking and research (at least as often as they can). Behavior and cognition can fruitfully be both studied and modeled, irrespective of whatever might be going on at the level of neurons, brain regions, and circuits, so why bother with the substrate? Given that this substrate happens to be the most complex object in the known universe, it may seem altogether appropriate to investigate higher-level cognitive and behavioral phenomena as a line of scientific inquiry that remains largely independent—if not completely divorced—from neuroscience. The intention here is not to accuse, but rather to acknowledge (1) that *all* viewpoints are limited, and (2) that neuro-agnostic cognitive scientists and psychologists may specifically benefit from a broader vantage that includes some degree of implementation-level understanding. To the point, researchers who are sympathetic to the ecological rationality approach should accept that a proper accounting of an animal’s structural organization and limitations requires an appreciation of its embodied particulars, including the evolved neural architecture and perceptual apparatus that underlie behavior and cognition in the animal and its conspecifics (at least to the extent that it may be practically relevant).

As a specific example of the potential usefulness of this embodied/embedded ecological rationality perspective, this paper argues in favor of a greater implementation-level awareness of cortico-basal ganglia-thalamo-cortical—or CBGTC—circuitry, sometimes referred to as the CBGTC loop (e.g., Parent and Hazrati, 1995). Critical to the regulation of goal-directed action selection in vertebrate motor control, the architecture of CBGTC circuitry (or its functional equivalent in species that lack a neocortex) has been conserved throughout the evolution of every vertebrate species (Reiner, 2010; Stephenson-Jones et al., 2011); equivalently fundamental to motor control and motor learning in each of those species, this circuitry allows individuals within the vertebrate lineage to both learn basic sequences of goal-directed actions and to successfully achieve their goals by recalling and executing those sequences in situationally appropriate contexts (e.g., Grillner and Robertson, 2016). Furthermore, this same neural circuitry is implicated in cognition (e.g., Graybiel, 1997; Middleton and Strick, 2000), including the production and comprehension of human language (e.g., Lieberman, 2002; Reimers-Kipping et al., 2011). The evidence suggests that this evolutionarily ancient circuitry evolved as an effective and efficient means for learning and regulating sequences of goal-directed motor behaviors, and that this functionality was extended over time *via* exaptation^1^ —at least in human evolution—to serve analogously in cognitive control, providing us with the means to learn and regulate sequences of goal-directed cognitive operations. The extent of this functional overlap between motor and cognitive control makes these circuits an attractive starting point for an expanded implementation-level awareness among neuro-agnostic students of behavior and cognition.

We begin the rest of this paper with a summary overview of the recurrent structure of CBGTC circuitry, as well as its relevance to the regulation of action selection and the coordinated sequencing of goal-directed action (e.g., Park et al., 2020; Dhawale et al., 2021); we then review the role of this circuitry in procedural learning and the development of action sequence protocols and (in some repeating contexts) the transition away from voluntary execution of those protocols and toward their automatic expression in response to contextual triggers—i.e., habit formation (e.g., Graybiel, 1995). Following that basic overview, we outline a symbolic model of these implementation-level processes, which provides a general framework for understanding and describing behavioral phenomena in terms of an embodied agent, its goals, and the ecological contexts that emerge between goal-directed action (*via* perception and motor control) and environmental structure; this model also provides a common language that helps illustrate the relevance of CBGTC circuitry for cognition by highlighting the functional overlap between motor control and cognitive control. We then apply this framework to heuristic decision-making and the “adaptive toolbox” (e.g., Gigerenzer and Todd, 1999b) and discuss how our model may benefit current thinking and future work in ecological rationality and other areas of cognitive science.

## A rough sketch of voluntary motor control and sequential goal-directed behavior

The neural circuitry of the CBGTC loop is complex, but it is not difficult to convey a simplified understanding of what the brain is doing during (and immediately prior to) voluntary action in the case of motor control. From the endpoint of the literal muscular activations that resulted in one of the authors typing on a keyboard, we can roughly trace the sequence of neural activation backward through the relevant circuitry to the initial intention to write the words you’ve just read (because all voluntary motor control invariably begins with a goal; for an accessible and less-physiologically-oriented overview of this process in greater detail, see Wong et al., 2015).

The motor cortex is ultimately responsible for the literal execution of voluntary movement *via* coordinated muscular activation; when a sentence is typed on a keyboard, it is because the appropriate somatotopic regions of the motor homunculus (M1) have been activated in order to move the muscles controlling the fingers just so, such that the goal of typing this or that word is ultimately achieved. This sequential, temporally coordinated pattern of activation is processed in premotor areas of the cortex (such as Broca’s area), but the finalized sequence is ultimately forwarded to M1 only after the relevant cortical regions have been stimulated by excitatory subcortical projections from the thalamus; before the relevant thalamic neurons may excite those proper cortical pathways through to M1 in that way, the thalamus must first be selectively disinhibited by the subcortical nuclei of the basal ganglia^2^. One role of the basal ganglia is to serve as gatekeepers of behavioral expression—generally inhibiting thalamic activation while selectively opening the “gates” (*via* targeted selective cessation of that inhibition) to permit specific thalamic excitation to occur—coordinating which behaviors are ultimately expressed (and when). Prior to the basal ganglia releasing their inhibitory grasp on the particular thalamic neurons that will go on to excite motor areas of the cortex, the basal ganglia receive input from the prefrontal cortex (and elsewhere) regarding the motor goal, a motor plan that is predicted to achieve that goal, and sensory input associated with perception of the current context (i.e., sitting/staring at the computer, working to complete a draft of this document).

To summarize this progression in its proper order, (1) prior to typing, an intention in the cortex—e.g., to type the word *cortex*—forms the basis of a motor goal that leads to the selection of a planned motor sequence—e.g., to move particular fingers in series over the keyboard—which is predicted to achieve that goal; (2) this information is then projected subcortically to the basal ganglia, which (3) sequentially disinhibit select regions of the thalamus that (4) will correspondingly excite the cortex, leading to the behavioral execution of the motor plan. Of course, how fluidly this progression unfolds depends on one’s prior experience/facility at typing. To type the same word, a student first learning to type may initially need to form distinct intentions, goals, and motor plans in order to press particular letter keys individually with specific fingers (and not others); however—over the natural course of procedural learning—the actions that achieve the lower-order goals of individually pressing the C-O-R-T-E-X keys may come to be sequenced together automatically when pursuing the single higher-order goal of typing the word *cortex*.

## Procedural learning and habit formation in vertebrates

In general, if the execution of a motor plan in some context successfully achieves the motor goal that inspired that plan’s initial selection, dopaminergic neurons provide reinforcement signals to the relevant sections of the CBGTC loop; this process of reinforcement forms associations that result in an increased likelihood of re-selecting that same motor plan in any future instance in which that same goal recurs within that same context (or similar contexts). When trial-and-error exploration is added, this combination of goal-directed motor control and reinforcement amounts to a basic description of procedural learning: Simpler behavioral elements that achieve lower-order goals are strung together (serially and/or in parallel) to form a more complex action sequence, which is executed in pursuit of a more complex higher-order goal (that the sequence is predicted to achieve); when a sequence of behavior achieves its goal, it is contextually reinforced in association with that goal and its concurrent/immediately preceding ecological features; the more frequently a given sequence achieves its goal and is reinforced in a consistent context, the deeper the association becomes between that goal, the sequence of behavior that achieved it, and other contextual features that consistently coincided with/preceded them—and the more consistently and efficiently that sequence is then selected and executed in the future when that constellation of reinforced associations subsequently realigns.

If the process of contextual and procedural reinforcement recurs consistently and frequently enough, the selection and coordinated execution of an action sequence may crystallize into a habit. In behavioral neuroscience, a habit describes a stereotyped sequence of goal-directed behavior that has become automatic^3^ through “overlearning” (i.e., through consistent repetition within a stable context): over the course of many trials, individual behaviors of a sequence gradually fuse together into a singular “chunk” of behavior that becomes associated with—and triggered by—its context (e.g., Graybiel, 1998, 2005; Smith and Graybiel, 2014, 2016). In other words, the associations between goal, behavior, and coincidental contextual cues eventually become so strong (under the right conditions), that perceiving the associated cues will trigger the entire sequence of behavior through to its completion at the achievement of the goal. A study by Barnes et al. (2005) provides a window into the neurological development of a habit within the CBGTC loop. For this experiment, rats were repeatedly placed in a simple T-shaped maze; as a rat approached the T junction, a tone from the left or right reliably signaled which arm of the maze the rat could follow in order to find a chocolate pellet reward (which it was allowed to eat, if it chose the correct arm). Initially, single-unit recording within the rats’ basal ganglia revealed a constant and chaotic pattern of activation that corresponded with the halting exploratory motion with which the rats first examined the maze; however, as the rats became accustomed to the structure of this task environment (over the course of many trials), the pattern of striatal activation changed as their behavior became more efficient and consistently successful: task-irrelevant neural activity dropped off drastically, and task-relevant firing clustered around the beginning and end of the task. After this period of overlearning, the rats entered an “extinction” phase of trials—in which the source of the tone no longer reliably indicated which arm of the T-maze contained chocolate—followed by a “reacquisition” phase that re-established the consistency between tone and reward; the rats’ neural activity reverted to initial levels of chaotic activation during extinction trials, but rapidly resumed pre-extinction firing patterns after the onset of reacquisition trials (Barnes et al., 2005).

The pre-extinction shift in activation reflects the general nature of procedural learning and (later) habit formation: What was once a series of distinctly-exploratory actions, executed individually in pursuit of multiple disjointed goals (e.g., *check over there*; *try forward and to the left*; *now right*; *ooh, eat this chocolate!*), becomes consolidated into a unified “chunk” of behavior, executed collectively in response to a set of contextual triggers that has become associated with that behavioral chunk and its achievement of a single, overarching goal. What was once an unfamiliar context—in which exploration *occasionally* resulted in a chocolate reward—has become a recognized context in which adherence to a strict behavioral protocol *always* results in a reward. A habit naturally starts to form as any vertebrate animal (e.g., a rat) experientially discovers that a recurrent goal (receiving the chocolate pellet upon solving a maze) is repeatedly achieved *via* the execution of a stereotypical sequence of behavior (following a direct route to the maze’s end, given a tone on one side) whenever it perceives that it has reencountered that context^4^. After a habit has become established, the perception of its associated contextual cues automatically triggers the onset of that habit, which runs through to its completion (whereupon the goal is achieved).

## The ecological context model: A formal account of embodied/embedded motor control

Generally, in the context of a desired goal in a particular environment, the process of procedural learning *via* trial-and-error exploration and reinforcement can be summarized as the construction (*via* motor control) of a novel action sequence that is discovered to be successful at achieving the desired goal (in that particular environment). In *recurrent* contexts—where a desired goal is repeatedly pursued in a particular type of environment that is stable enough to support the reuse of stereotyped behavior over the course of repeated encounters—the processes of procedural reinforcement (and habit formation) can be summarized as streamlining the selection of a sequence of actions that consistently achieves its goal in the associated environments (and the consolidation of that sequence into a singular behavioral chunk). Given this basic understanding, we can roughly characterize how vertebrates physically navigate their environments and pursue their goals, flexibly stringing simpler behaviors together into more complex sequences in an exploratory fashion, using trial-and-error learning to discern which sequences achieve their goals, and—in recurrent contexts—refining behavioral protocols and developing habits to efficiently and effectively exploit stable (i.e., predictable) environmental structure.

From here, we establish a symbolic description of what occurs in these phenomena, which might be considered a generalized extension of Lewin’s (1936) field theory equation in which behavior *B* is expressed as a function of the interactions between a person, which we will generalize to an agent *A* and its environment *E* as such:


B=f⁢(A,E).

While Lewin’s equation importantly entails that an agent’s behavior necessarily depends on the ecological interactions between that agent and its environment, the nature of the function and the particulars of *A* and *E* are unspecified (Todd and Gigerenzer, 2020).

Rather than starting with behavior, we begin with an ecological context *C*, which refers to the unique situational configuration that arises when an individual agent *A* is oriented toward a specific goal *g* within a particular local environment *E*, as follows:


C={A⁢(g),E},

where—for a given context—the environment *E* consists of a set of structural features, where


$$E=\left\{f_{1},f_{2},f_{3},\ldots ,f_{n}\right\},$$

and *E* contains an agent-dependent subset— *E*(*A*)—consisting of structural features that are hypothetically perceptible by the agent, depending on its perceptual apparatus^5^. The agent *A* possesses a given repertoire of possible behaviors *Br*—whether learned or innate—where


$$\mathrm{Br}=\left\{b_{1},b_{2},b_{3},\ldots ,b_{n}\right\}.$$

The agent *A* also possesses a set of recent (including current^6^) perceptions *P*, following from phenomenal awareness/experience of some perceived subset of *E*(*A*), where


$$P=\left\{p_{1},p_{2},p_{3},\ldots ,p_{n}\right\},$$

and *A* similarly possesses a set of related behavioral associations *Ba*—given *g* and *P*—where


$$\mathrm{Ba}\,\left(g,P\right)=\left\{b_{1}\,p_{1}\,g,b_{1}\,p_{2}\,g,b_{2}\,p_{1}\,g,\ldots \right\}.$$

Based on *Ba*, the agent plans and executes a behavior or sequence of behaviors *B*, drawn from *Br*, where (for example)


$$B=\left[b_{i},b_{j},b_{k},\ldots \right],$$

and *B* is predicted to achieve *g*; if that prediction is successful and *g* is achieved—*g*^+^—following *B*, then *B* is reinforced, and its related associations in *Ba*(*g*,*P*) are updated, such that *B* is then more likely to be executed in a similar context— *C*′—in the future, where *g* recurs in *C*′ and there is overlap between the agent’s perceived features in *P*(*C*) and *P*(*C*′).

In keeping with the themes of embodiment and embeddedness, the agent is only nominally separate from the environment in this model out of convenience: *E* and *E*(*A*) should be understood to contain features that are internal to the agent as well as external—e.g., *E* includes the agent’s cognitive architecture, behavioral repertoire, circadian rhythm, etc., and the agent-specific perceptible subset of *E*, *E*(*A*), includes the agent’s memories, emotions, interoceptions, and any other internal characteristics or processes that might enter its phenomenal awareness.

If any element in the configuration of a given ecological context is altered, that new configuration necessarily entails a different context. Because the set of an agent’s perceptions, *P*, includes perceptions in the present moment, this might occur (for example) if the agent is unexpectedly interrupted and reorients toward a new goal (e.g., upon the arrival of a potential mate); or this may occur if the agent returns to a familiar and unchanged environment with an expanded or reduced behavioral repertoire (e.g., subsequent to learning or a restricting injury, respectively); or if the environment has changed (even imperceptibly—e.g., a trap has been set and thoroughly hidden); etcetera. And although it may seem unwieldy to differentiate between ecological contexts, given even the slightest changes to their constituent elements, this practice highlights the primacy of goals and their associations: the cyclical recurrence of goals (e.g., the periodic importance of the goals to eat and drink, inspired by oscillations in hunger and thirst) connects contexts over time, allowing agents to discover and exploit structural invariance across those contexts (e.g., by repeatedly returning to the location of a reliable watering hole to drink); an extended description of an agent/environment system over time can be characterized as a succession of contexts, depending on the prevailing goal of the agent within a given moment.

To organize and summarize, an ecological context can be expressed as comprising an agent’s orientation toward a specific goal in its present environment,


(1) C={A⁢(g),E},

where an agent’s behavior in a given context can be expressed as a function of its behavioral repertoire and its behavioral associations (given its present goal and recent/current phenomenal awareness/perception within that context),


(2) B⁢(C)=f⁢(Br,Ba⁢(g,P)),

and where the achievement of a goal in a given context can be expressed as a function of an agent’s behavior and the structure of its environment,


(3) $$g^{+}\,\left(C\right)=f\,\left(B,E\right).$$

This degree of formalism allows us to systematically characterize a wide range of observed behavioral phenomena in terms of their associated contexts and contextualized interactions.

### Procedural learning and habit formation in the ecological context model

Within the framework of this model, we can describe the characteristic progression from trial-and-error-based exploration through procedural learning and habit formation as a transition through a series of ecological contexts—following (1)—in which *g* and the external structure of *E* are held constant. In early contexts, the agent’s expressed behavior—following (2)—is exploratory and unpredictable, but as *Ba*(*g*,*P*) is updated (*via* reinforcement), later contexts in the series become more and more autocorrelated as behavior under (2) converges upon a stereotyped protocol that consistently achieves *g* under (3), given the fixed external structure of *E*—i.e., after a point, the outcome of (3) becomes predictable for all subsequent contexts in which the relevant structural features^7^ of *E* and the perceived features *P* are effectively stable. When *B*(*C*) becomes “chunked” into a single behavior (as occurs in habit formation), it is considered to have been added to the agent’s behavioral repertoire, such that


$$B\,\left(C\right)=b_{n+1},$$

and where *b*_*n* + 1_ has been appended to the set *Br*, and may subsequently be recruited (à la transfer of learning; e.g., Day and Goldstone, 2012) in new behavioral sequences—following (2)—potentially in pursuit of unrelated goals in different contexts (e.g., during future trial-and-error exploration).

This framework may similarly be used to illuminate how and why habits occasionally break down and result in error. When an individual who typically drives their own car habitually attempts to shift from PARK into DRIVE in an unfamiliar rental, this will often result in the individual grasping a fistful of air (instead of the shifter) if it happens to be located behind the steering wheel rather than its accustomed spot in the center console of the familiar car (or vice versa); this can readily be understood in terms of preparing to drive in the familiar context of the known car *C*, and preparing to drive in the different, but structurally similar context of the unfamiliar rental *C*′. In both contexts, the goal *g* (to shift from PARK into DRIVE) is the same, and overlap in perceived features across *P*(*C*) and *P*(*C*′) is sufficient to trigger the habitually-chunked sequence of behavior *B* in both contexts, following (2); however, structural differences between *E*(*C*) and *E*(*C*′) are such that the habit fails to achieve *g*^+^ in *C*′ where it is consistently successful in *C* [following (3)]. After the failure, *g* persists unachieved in *C*′, which typically motivates visual exploration to update *P*′—i.e., to perceptually locate the shifter—followed by the formation and execution of an adjusted motor plan *B*′ that is predicted to achieve *g* and which might (given consistent repetition and reinforcement) become a new habit in *C*′ if the unfamiliar car is driven frequently enough over a sufficient period of time.

This descriptive model was designed primarily to provide a conceptual pivot point—to facilitate a shift in discussion from motor control in vertebrates, generally, to cognitive control in humans, specifically. Given its ubiquity in vertebrates, the functional architecture of CBGTC circuitry is extremely well-studied (e.g., Foster et al., 2021). To reiterate, the same functional circuitry in humans is found even in the relatively simple lamprey, which diverged from the rest of our ancestral vertebrate line ∼560 million years ago: The basal ganglia in lamprey brains perform the same role in action selection as they do in modern humans, inhibiting most behavior but selectively disinhibiting actions in sequence to achieve specific motor goals (Grillner and Robertson, 2016). This suggests that the basal ganglia evolved (at least in part) to facilitate action selection in a pre-vertebrate species, and they were so effective that they remain virtually unchanged among all vertebrate species over half a billion years later (Reiner, 2010). This evidence strongly suggests that all vertebrates use the same CBGTC circuitry (or its functional equivalent) to orchestrate the timing and sequencing of motor actions—selected from among a general repertoire of possible actions—in the pursuit of various motor goals (Stephenson-Jones et al., 2011). Moreover, evidence *also* suggests that humans use CBGTC circuitry to orchestrate the specific timing and sequencing of *cognitive* actions—also selected from among a general repertoire of possible operations—in the pursuit of various cognitive goals (e.g., Lieberman, 2007; Graybiel, 2008). The next section formulates some decision-making research in terms of the ecological context model framework, to highlight the significance of this circuitry for cognition, and to demonstrate the potential benefits of viewing decision making and other cognitive phenomena through the lens of this particular embodied and embedded perspective.

### Heuristics and the adaptive toolbox in the ecological context model

As traditionally conceived in the ecological rationality literature, a heuristic is an algorithmic process that uses limited environmental information in order to make effective and efficient decisions, assuming that the structure of the task environment appropriately matches the heuristic (Gigerenzer and Todd, 1999a). For instance, the *elimination-by-aspects* heuristic is a choice-making algorithm that first compares the available options on the basis of a single cue: If one option outscores the rest on that criterion, that is the choice—but if no option outscores any others on the basis of that cue, the sample of options is potentially reduced, the next cue is selected and checked to see if it determines a unique choice, and the process repeats down the line of possible cues until a choice is made (Tversky, 1972). If you are in a new town and need to decide on a restaurant to visit for dinner, you could be an unagi fan and pick sushi as your first cue, but then find that 3 out of 14 nearby restaurants serve sushi, so you limit the field to those 3, use price as your next cue, check out their menus, and then make your choice based on which spot offers unagi for the best price. This heuristic works well across various contexts, making an efficient choice in environments where options differ on a range of attributes.

Within the formal system outlined previously, we can reframe such heuristic decision-making algorithms like this in such a way that they are rendered indistinguishable from the context-sensitive execution of refined motor sequences and habits as described above. Just as habits, a heuristic can be represented in terms of this framework as a sequence of goal-directed cognitive operations—a decision mechanism selected (from among others in an existing repertoire) because it is expected to achieve a specific goal in a given ecological context. In the case of *elimination-by-aspects*, we would predict that this heuristic would be likely to be selected for use in any context in which the goal *g* is to make a choice in a decision-making environment *E* and in which three assumptions are met: First, that its perceptible features *E*(*A*) include multiple choice options with discernibly (or conceivably) differentiating attributes—following (1); second, that the perceived features of that context, *P*, overlap with perceived features in prior contexts in which expressed behavior—following (2)—was the *elimination-by-aspects* heuristic; and third—following (3)—that the use of this heuristic resulted often enough in the achievement of *g* when it was deployed in similar contexts in the past.

When reframed in this manner, heuristic-based errors may also be rendered formally indistinguishable from habit-based errors, such as in the above example of the habitual shifter-grasping error that sometimes occurs when driving an unfamiliar car. As indicated above, grabbing at the air above a rental car’s center console can be formulated as an instance in which habitual behavior is erroneously triggered in an unfamiliar context because it shares a goal and has overlapping features with a familiar context (in which the habitual behavior has previously been effective); in this case, behavior that would have been successful in one context leads to failure in the other, because the environmental structure of the second context is incompatible with goal achievement, given that behavior, as per equation (3). This can be seen to parallel successes and failures in the case of heuristic decision making.

For example, consider use of the *recognition heuristic*: Roughly, when facing a decision between two options wherein one is recognized and the other is not, choose the recognized option. Goldstein and Gigerenzer (2002) showed that students tend to use the recognition heuristic when asked which of two cities is more populous; the recognition heuristic is often successful in the context of questions like this, because cities that are larger tend to be more famous—hence more often talked about and consequently more recognizable—than smaller cities (Todd, 2007). But consider how American students would likely fare (on average) if asked which city is more populous in two different contexts: (a) comparing Japan’s two largest cities, Tokyo and Yokohama, and (b) comparing Yokohama and Nagasaki, which is toward the bottom of the top 50 most populous Japanese cities. In both contexts, the goal is the same, as are the perceptible features, so we would predict (absent explicit individual knowledge) the use of the recognition heuristic in each instance, following (2). Consequently, because Tokyo and Nagasaki are likely both highly recognized (relative to Yokohama), Tokyo would likely be (correctly) chosen in the first comparison, and Nagasaki would likely be (incorrectly) chosen in the second. From the perspective of the ecological context model framework laid out above, this occurs because the underlying structure of the first context supports the recognition heuristic’s successful use, following (3), but the structure of the second context is incompatible with that heuristic (while being similar enough to compatible contexts in order to elicit it).

The points of similarity between habits and heuristics suggest the possibility that there is little difference between them (at least in terms of their formation and implementation, according to our model). If this is correct, it would imply that at least some—if not many or most—heuristics in the adaptive toolbox have likely been formed in the same way that procedural memories and habits form: individually, *via* trial-and-error-based procedural learning, by combining available operations in pursuit of a specific goal in the context of a particular environment. In the last section of this paper, we argue that this is likely the case—namely, that the evolutionarily conserved circuitry that underlies vertebrate motor control has been coopted to facilitate the use of cognitive control to pursue and achieve cognitive goals analogously to how motor goals are pursued and achieved *via* motor control.

## Goal pursuit in motor and cognitive domains: Evidence for generalized implementation

In general, if a description of a cognitive phenomenon—like a heuristic—can be expressed in terms of a sequence of actions or operations that are executed in pursuit of an identifiable goal in some context(s), we suspect it is a reasonable first assumption that—at an implementation level—the phenomenon in question relies on CBGTC circuitry (or its functional equivalent, as is the case for any overt sequence of goal-directed motor behavior in every species of vertebrate). Some of the advantages that this approach may bring to the study of cognition can be appreciated in terms of its analogous success in previous research comparing internal and external search (e.g., Hills et al., 2008, 2015a; Todd and Hills, 2020). This work suggests that search behavior in both physical and cognitive domains likely relies on a shared set of underlying neural mechanisms, and that these mechanisms almost certainly first evolved to facilitate exploration through external space and subsequently (much later) were further adapted *via* exaptation to similarly regulate exploration throughout internal space as well. Whereas cognitive search is possibly unique to humans (and its observation is relatively obscured by our skulls), physical search is practically ubiquitous in the animal kingdom (and is relatively straightforward to observe); given their shared implementation and common evolutionary provenance, a well-developed understanding of the nature of physical search—which is relatively easy to attain—can serve as a natural source of valuable insight into the nature of cognitive search (Hills et al., 2015b; for general discussion, see Todd and Miller, 2018).

Akin to the ubiquity of search, much of what we and other animals *do*, both in terms of our behavior and our cognition—including exploration, communication, and decision-making—amounts to the pursuit of various types of goals within various types of environments. Ultimately, it appears that CBGTC circuitry allows for specific behaviors and/or cognitive operations to be pieced together (serially and in parallel) into goal-directed sequences, to recognize when specific sequences are rewarding in particular contexts (because they achieve their associated goals), and to consolidate or “chunk” sequences of rewarded actions into singular protocols (which themselves may then be recruited in other contexts to create even larger sequences in pursuit of more complex goals, eventually contributing to the formation of even larger chunked protocols). Given this perspective of CBGTC circuitry as a kind of recursive^8^ sequencing engine that constructs, executes, and evaluates the efficacy of goal-directed action patterns, it appears possible (if not likely) that its functional architecture is so highly conserved among vertebrates precisely (or at least in part) because of how successfully it regulates the embodied pursuit of goals and the learning of embedded goal-pursuit protocols that are custom-molded to fit and exploit structural regularity in the environment wherever possible.

To adopt this neurologically-grounded perspective of heuristics as goal-directed behavior/cognition is to explicitly connect ecological rationality to the common neural architecture that is responsible for orchestrating goal-oriented motor behavior in vertebrate brains. This has the immediate benefit of simplifying the *strategy selection problem* (e.g., Marewski and Link, 2014)—in which the mechanism for choosing a given heuristic or strategy in any given context is unspecified and difficult to implement artificially—as the answer to this problem reduces to the analog of the combined processes of trial-and-error-based procedural learning, context recognition, and goal-directed action selection in vertebrate motor control (which are relatively well-studied in non-human vertebrates). Additionally, this view emphasizes the primacy of specific goals in behavior as well as in cognition. There is often an implicit generalization and abstraction of goals when “rationality” is defined traditionally in terms of “human reasoning” whereby “to act optimally” or “to make an optimal decision” could effectively characterize the presumed goal in any given situation. In contrast, the ecological context model that we propose encourages a perspective of rationality that is relative to an embodied agent’s pursuit of its own *specific* goal (or set of goals) within the environment in which it is embedded; in this view, rationality—with respect to some agent’s behavior—must be conceptualized and evaluated in terms of the agent’s goal(s) that gave rise to the behavior in an environment, and whether the behavior in question was successful—with respect to the agent’s goal(s)—in that context.

Ultimately, the ecological context model is a conceptual framework that may inform a range of approaches to interdisciplinary scientific inquiry. Tying goal-directed cognition to the neurophysiology of goal-directed motor control constrains the possible ways in which goal-directed cognition may have emerged during the course of evolution. This suggests that evolutionary theorists may gain insight by developing a greater understanding of the normal functioning of CBGTC circuitry in non-human vertebrates (e.g., Desrochers et al., 2010), the cognitive and behavioral consequences of its malfunctions in related pathology (e.g., in cases of Huntington’s, Parkinson’s, and FOXP2 mutations), as well as the abnormal behavior and anatomy/physiology of CBGTC circuitry in non-human animals that have been reared with humanized^9^ genes that affect the development of that circuitry (e.g., Schreiweis et al., 2014). Further, neuroanatomists and behavioral neuroscientists may uncover new insights by investigating structural and functional differences in CBGTC circuitry across humans, non-human primates, and other mammals. Moreover, as CBGTC circuitry is so functionally conserved in vertebrate motor control and motor learning, psychologists and cognitive scientists may themselves derive new insights from existing work in neuroanatomy and behavioral neuroscience relevant to CBGTC circuitry: Some questions about hypothetical mechanisms of human cognition may become simpler when plausibly grounded by comparisons to potentially-corollary mechanisms in vertebrate motor control/learning that are already relatively well-studied in non-human animals.

## Author contributions

SN wrote the first draft of the manuscript. Both authors contributed to manuscript revision, read, and approved the submitted version.

## Footnotes

1 Exaptation is the co-opting or repurposing of existing structure/functionality over the course of evolution by natural selection—a process by which pre-evolved structure/functionality is subsequently further adapted or co-opted, extending its use or operation to fit new modes or contexts for which it was not originally adapted (e.g., Gould, 1991; for relevant discussion on exaptation in the context of rationality, see Mastrogiorgio et al., 2022).

2 Technically, the relevant areas of the thalamus are always “attempting” to excite the cortex, but normally they are reined in by tonic inhibitory input from the basal ganglia, which persists until selective disinhibition allows targeted thalamic excitation to stimulate the cortex in a controlled fashion—this is why pathology of the basal ganglia can either lead to a chaotic excess of unintended movement (as in Huntington’s chorea) when generalized inhibition falters, or deficits in voluntary motor control (as in Parkinson’s disease) when selective disinhibition is impaired.

3 Roughly in the dichotomous sense of automatic vs. controlled processing (Schneider and Shiffrin, 1977).

4 Evidence supports a kind of retrograde contextual expansion in the development of a habit. A habit’s endpoint is naturally tied to the achievement of its associated goal, but the neurological markers of a habit’s onset apparently may shift backwards in time (relative to achieving the goal) in a way that reflects an updating of when/where that habit’s context begins (effectively enlarging the “chunk”). Barnes et al. (2005) report that these neurological-onset markers for their rat’s habits were originally recorded around when experimental trials began as the maze door opened and the rats entered the maze; however, over the course of further trials, these markers began to occur earlier and earlier in time, with recorded activation eventually settling around when experimenters first placed the rats into the pre-trial antechamber (where they waited for a few moments before the maze door opened and trials “officially” began). This suggests that habits are constructed in reverse for cases in which the structural stability (i.e., invariability) of a recurrent context supports the use of a stereotyped behavioral protocol to achieve a goal, and that the protocol expands to match the temporal/structural invariability of its context.

5 For example, the presence and reflectance of ultraviolet light would always be considered structural features in a given environment, but they would not normally be perceptible features for humans (absent special tools) in the way that they would be for most birds.

6 This ecological context model is organized around a single goal and its pursuit, to keep it simpler, but there may be multiple competing goals that are simultaneously “vying” to be pursued in any given moment (or which might be pursued in tandem), and the particular goal that takes precedence may change from moment to moment. The model could be adjusted to better capture continuous-time dynamics by changing the singular goal to a set of goals with associated motivation levels that fluctuate in response to real-time perceptions and/or changes to internal and external environmental factors (e.g., a function of rising hunger over time would increase the motivation to seek food, or the sudden appearance/perception of a dangerous predator would cause a spike in the motivation to fight and/or flee, eventually or suddenly leading to a shift in goal orientation that would entail the formation of a new ecological context in the model), which would govern transitions between contexts.

7 Relevant in the sense of being integral to the successful execution of the behavior—e.g., the color of two otherwise identical cars is irrelevant to driving behaviors, but relevant to trying to locate one in a parking lot.

8 Recursive in the sense used by Hauser et al. (2002), who characterize recursion as “the capacity to generate an infinite range of expressions from a finite set of elements” (p. 1,569).

9 For example, mice may be reared with the two amino acid substitutions that differ between the human FOXP2 gene and the mouse Foxp2 gene (Enard et al., 2009).
